# Supplementary material for: Investigating the mitochondrial genomic landscape of Arabidopsis thaliana by long-read sequencing
Source: PLoS Comput Biol. 2021 Jan 12;17(1):e1008597. doi: 10.1371/journal.pcbi.1008597 (PMC7833223; doi:10.1371/journal.pcbi.1008597)
Supplement: Attachment — Submitted filename: QandA_2nd.docx [file pcbi.1008597.s001.docx]

We are grateful to the two reviewers for their useful and insightful suggestions. We have revised the manuscript accordingly. Let us describe our responses to individual comments on what follows.

Reviwer:1

***Comment***: Title: To my liking, talking about ‘plants’ when you only study a particular group of plants, in this case only one species, it’s rather pretentious. I know it’s something that is used by many authors, but I do not agree with this. I would change to a more appropiate title that describes the group of plants that it’s used in the study.

***Answer***: We thank the reviwer1 for their insightful comments. We agree with the reviewer’s opinion and changed our title to “Investigating the Mitochondrial Genomic Landscape of *Arabidopsis thaliana* by Long-read Sequencing.”

***Comment:***
Abstract 1. Edit ’is not easy.’ for ‘is not feasible.’ 2. Edit: ‘Specifically, to construct a sensitive read classifier, we developed a method that exploits two classic algorithms--partial order alignment (POA) and the hidden Markov model (HMM).’ for ‘We developed a method that exploits two classic algorithms--partial order alignment (POA) and the hidden Markov model (HMM) to construct a sensitive read classifier.’

***Answer:*** We have revised these sentences accordingly.

***Comment:***
3. Edit ‘a 15% sequencing-error rate and produced highly correct clustering.’ for ‘a 15% sequencing-error rate and produced correct clustering.’ It is my believed that something is correct or not, you cannot state that something is highly correct.

***Answer***: We agree with the reviewer’s comment and revised the sentence as ‘a 15% sequencing-error rate and produced accurate clustering.’ This is because, as we observed some misclassified reads, the word ‘correct’ was not appropriate.

***Comment:*** 1. Edit: ‘For this prepose’ 2. Edit: ‘unknown structure of plant mitochondrial genome’ for ‘unknown structures of plant mitochondrial genomes’
Line 12: Edit: ‘Illumina sequencers’ for ‘Illumina sequencing technology’

***Answer:*** We corrected the misspelling and terminology.

***Comment***: Lines 34-49: The last two paragraphs of this section must be exchanged for a better organization. Minor edits must be done to accomplish this.

***Answer:*** We exchanged these two paragraphs and revised all sentences.

***Comment:*** Materials and Methods
Line 51: Eliminate ‘To begin’
Line 72 Edit ‘longest reads’ for ‘longest read’
Line 80 Edit: ‘shows the workflow so far’ for ‘shows the initial steps of the workflow’
Line 121: ‘POA graph to a yet another model’ for ‘POA graph to another model’
Line 124 Edit ‘introduce’ for ‘introduced’
Line 127: Edit ‘let us substitute’ for ‘we substitute’

***Answer:*** We revised our manuscript accordingly.

***Comment:*** Fig. 1. Legend
1. Edit: ‘The thik line’ for ‘The thick line’
2. ‘The thik line and thin lines represent a reference and long reads, respectively’ This is confusing in the figure. Please label which is the reference and which are long reads in the figure.
3. LU and U should be defined in the legend.

***Answer:*** We revised our manuscript and figure.

***Comment:*** Results
Fig. 2. Legend
1. Edit ‘We sampled 40 reads and 160 reads from one template and the other, respectively’ for ‘ We sampled 40 reads from one template and 160 reads from the remaining template.’

***Answer:*** We revised the sentence as suggested.

***Comment:*** Line 228: Edit ‘Results’ for ‘Performance’
Line 239: Delete ‘the’
Lines 240-241: Delete’As can be seen’
Line 246: Edit ‘clusters’ for ‘cluster’
Line 249; Edit ‘dedicated’ for ‘designed’
Line 254: Delete ‘Similarly,’
Line 262: Edit ‘more time’ for ‘longer’
Line 267: Edit ‘Results’ for ‘Performance’

***Answer:*** We have corrected these misspells and unnecessary words.

***Comment:*** Fig. 3. Legend
1. In (D), edit ‘other’ for ‘remaining’
Line 294: Delete ’For example’
Line 298: Delete ‘Specifically,’
Line 319: Edit ‘we speculate that the Kyoto stain’ for ‘it seems that this strain’
Line 320: Edit ‘and this structure is maintained within the strain’ for ‘and this structure has been maintained’
Line 326: Edit ‘speculate’ for ‘believe’
Line 325: Edit ’by means such’ for ‘by processes such’

***Answer:*** We revised our manuscript accordingly.

***Comment:*** Conclusion
Please discuss if your method would have any application for polyploid assemblies, specially autopolyploids.

***Answer:*** We appreciate this comment and add a short discussion about possible application for polypoid phasing. Specifically, we add the following sentences in the discussion:

*Another possible application of our algorithm is polyploid phasing of long-read. However, the current implementation is not designed for genome-scale datasets and runs too slowly for use with such large datasets.*

*We suspect that using multiple sequence alignment methods in place of the POA approach may provide much faster performance.*

***Comment:*** Line 338: Edit ‘However, there is room for improvement.’ for ‘There is room to improve our suggested method.’
Line 342: Delete ‘Also,’
Line 347: Delete ‘Lastly,’

***Answer:*** We have revised our manuscript accordingly.

Reviewer 2:

***Comment:*** The manuscript addresses an interesting problem; different from the mitochondrial genomes of animals, those of plants exist as multipartite structures that are difficult to assemble and describe using only short-read sequencing methods. The manuscript addresses this problem by proposing a new method to cluster long reads using a K-means algorithm that uses a HMM and partial order alignments to assign each long read to a different cluster. In general, the algorithms are described with sufficient detail both in the text and by using pseudocode. The underlying data and code are available from Github.
***Answer:*** We thank the reviewer for deeply understanding our manuscript and giving us insightful comments.

***Comment:*** The authors might need to add some extra information to the legend of Figure 1, which is somewhat vague, and need to explain better how they choose a particular value for K (the pre-defined number of clusters), as the number of existing structural variants is a priori unknown and might be very high.

***Answer:*** We thank for the reviewer for pointing out this issue. We add some notes on U and UL in the legend of Figure1.

Speaking of the number of the clusters, denoted by K, we first mapped reads supporting structural variations to reference sequences. Then, we checked if reads supporting different structures were present in the same section of the reference. Since there were very few such reads, we used K=3 as the default setting.

In other words, we presume that, in each section of the reference, there would be a major structure and a sub-structure (if any) for clustering; we know that there can be three clusters at locations such as the arrows in Fig 3.a, so we set K=3.

We should have made this assumption clear in the first version of the manuscript. Following the comments, we have inserted the following sentence at the beginning of the results section:

*After manual investigation of the reads indicating structural variations in the reference genome, we presumed that one minor component was present in each section of the reference in addition to the ``master circle.''*

*Therefore, we set the cluster number for each section ($K$) to $3$ and decomposed the reads from nine A. thaliana datasets.*

***Comment:*** The manuscript might also benefit from some additional details (e.g. details on the scoring scheme used in the POA dynamic programing algorithm, or information on the starting coordinates for the back-tracing in the same algorithm, considering that the authors follow a semiglobal approach to not considering erroneous trailing nodes).

***Answer:*** We agree with the reviewer’s comment and add some notes on the scoring schemes and strategy for back-tracing. Specifically, we add the following sentence to explain scoring scheme:

*In the result section, we set the insertion and deletion penalty to -3, mismatch penalty to -4, and match score to 3*

And next sentence to explain back-tracing strategy:

*Specifically, given we follow semi-global alignment approach, we first set i=arg max_i S[i][m], j=m and update i and j to the values giving the maximum value of the right-side of Eq 1 until j = 0.*

***Comment:*** Overall, I think the manuscript contains some interesting new ideas and tools to deal with the problem of cataloguing the different structural components of mitochondrial genomes in plants, a problem that is attracting much attention in the recent literature and is still waiting for solutions.
***Answer:*** We appreciate that the reviewer clarifies the purpose of our study and gives us useful comments.

***Comment:*** I also came across some typographical and other minor errors that the authors may want to fix:
* In the Author summary:
- it contains -> they contain/their genomes contain
- prepose -> purpose
- Appling -> Applying
- the last sentence in this section needs to be fixed too.
***Answer:*** We have revised these typographical and minor errors in our manuscript.

***Comment:**** Main paper:
-line 7: "a gene" is a vague expression; the authors might want to be more precise here.
- l. 8: create -> produce
-l. 51: may useful -> may be useful
-l. 75: LU? Please clarify.
- legend of figure 1: thik -> thick
- l. 102: how is the threshold selected?
l. 124: introduced
***Answer:*** We thank the reviewer for pointing out ambiguous sentence in our manuscript. We address the way we clean up the partial-order-alignment graph as follows:

*For computational efficiency, we removed erroneous edges during construction of POA graph. To this end, we recorded each edge's occurrence* $e_{i}$ *and removed erroneous edges with weight less than* $\tau\times\max_{i} e_{i}$*(*$\tau=0.4$ *by default) when the number of nodes reached a threshold (*$1.5\times$*maximum length of the input by default).*
